# Developmental dynamics of gene expression and alternative polyadenylation in the *Caenorhabditis elegans* germline

**DOI:** 10.1186/s13059-017-1369-x

**Published:** 2018-01-24

**Authors:** Sean M. West, Desirea Mecenas, Michelle Gutwein, David Aristizábal-Corrales, Fabio Piano, Kristin C. Gunsalus

**Affiliations:** 1 Center for Genomics & Systems Biology, Department of Biology, New York University, New York, NY 10012 USA; 2 Center for Genomics & Systems Biology, NYU Abu Dhabi, P.O. Box 129188, Saadiyat Island, Abu Dhabi United Arab Emirates

## Abstract

**Background:**

The 3′ untranslated regions (UTRs) of mRNAs play a major role in post-transcriptional regulation of gene expression. Selection of transcript cleavage and polyadenylation sites is a dynamic process that produces multiple transcript isoforms for the same gene within and across different cell types. Using LITE-Seq, a new quantitative method to capture transcript 3′ ends expressed in vivo, we have characterized sex- and cell type-specific transcriptome-wide changes in gene expression and 3′UTR diversity in *Caenorhabditis elegans* germline cells undergoing proliferation and differentiation.

**Results:**

We show that nearly half of germline transcripts are alternatively polyadenylated, that differential regulation of endogenous 3′UTR variants is common, and that alternative isoforms direct distinct spatiotemporal protein expression patterns in vivo. Dynamic expression profiling also reveals temporal regulation of X-linked gene expression, selective stabilization of transcripts, and strong evidence for a novel developmental program that promotes nucleolar dissolution in oocytes. We show that the RNA-binding protein NCL-1/Brat is a posttranscriptional regulator of numerous ribosome-related transcripts that acts through specific U-rich binding motifs to down-regulate mRNAs encoding ribosomal protein subunits, rRNA processing factors, and tRNA synthetases.

**Conclusions:**

These results highlight the pervasive nature and functional potential of patterned gene and isoform expression during early animal development.

## Background

Spatio-temporal regulation of gene activity is critical for the development of multicellular organisms and occurs at multiple levels. Post-transcriptional regulation influences messenger RNA (mRNA) processing [1], stability [2], localization [3, 4], and translation [5–8]. An mRNA’s fate depends on internal cis-regulatory sequences that are most commonly located in their 3′ untranslated regions (3′UTRs), and mutations within these have been associated with cancers and other diseases [9]. Functional elements in transcripts are recognized by trans-acting factors, including both RNA binding proteins (RBPs) and small regulatory RNAs [10, 11]. In the *C. elegans* germline, a complex network of RBPs and microRNAs controls germ cell proliferation and differentiation by regulating the expression patterns of numerous genes through their 3′UTRs [5, 12–15].

Germ cells are produced in assembly-line fashion in the gonad, progressing from mitotic proliferation in the distal region through meiosis and differentiation into oocytes and sperm in the proximal region. This developmental program depends on the spatio-temporal restriction of RBP activity, which is mediated largely by translational control of their own mRNAs through their 3′UTRs [14, 16, 17]. For example GLD-1, which is involved in the transition from mitosis to meiosis and oocyte differentiation [18–20], binds multiple targets [21] to both repress translation [22] and stabilize transcripts [17, 18, 23]. Translation of GLD-1 mRNA is negatively regulated by another RBP, FBF-1, which promotes mitotic proliferation and represses transcripts required for meiotic entry [24, 25]. Thousands of maternal transcripts are deposited into the embryo [26, 27], where a combinatorial code of translational regulators controls key developmental patterning events [8, 17, 28, 29].

The selection of cleavage and polyadenylation (CPA) sites during mRNA processing determines the sequence content in 3′UTRs, and thus the landscape of cis-regulatory elements available to trans-acting factors. Since longer 3′UTRs have more regulatory potential than shorter 3′UTRs, alternative polyadenylation (APA) can have significant functional consequences [30, 31]. 3′UTR variation during development and in different cell types is most commonly achieved by regulated selection of proximal or distal APA sites downstream of a shared terminal coding region, but can also affect coding sequences [31–39]. About half of genes in yeast, plants, worms, flies, zebrafish, mice, and humans produce multiple 3′UTR isoforms through APA, indicating that regulated isoform selection is a deeply conserved and widespread gene regulatory mechanism [34, 37, 40–47].

The production of alternative 3′UTR isoforms is coordinated with cell state and is influenced by cell-type-specific differences in chromatin structure, epigenetic marks, transcriptional rates, splicing and 3′-end processing factors, and competitive binding of regulatory RBPs [31, 48]. Differentiated cells, and neurons in particular, tend to display longer 3′UTRs [36], and progressive shifts toward longer 3′UTR lengths are observed during development in both worm and mouse [35, 43]. Conversely, proliferating cells generally have shorter 3′UTRs [32] and inducing pluripotency correlates with 3′UTR shortening [49]. In *C. elegans*, genome-wide 3′UTR studies have documented widespread APA in different developmental stages [43, 44] and tissues [38, 39]. The body of evidence thus suggests that target site accessibility for factors that recognize functional elements in 3′UTRs is an important regulatory platform for mRNAs that is integrated into developmental programs controlling specialized cellular functions [50, 51].

Because post-transcriptional regulation of maternal mRNAs plays a key role in the *C. elegans* germline and early embryo [8, 16, 17, 29, 52, 53], we hypothesized that APA may serve as a mechanism to modulate gene expression between progressive cellular stages within the gonad. However, previous studies have lacked the resolution to examine transcriptome-wide spatio-temporal dynamics during gametogenesis [27, 32, 43, 54–57]. Here, we present a map of 3′UTR isoform expression across different regions of the adult hermaphrodite germline, based on a novel method we developed that quantifies transcript levels and precise 3′ termini using small samples of RNA. By analyzing 3′UTR isoforms in dissected samples, we characterize the dynamics of gene expression and APA throughout gametogenesis and demonstrate isoform- and cell-type-specific variation between sexes and within distinct regions of the female germline. We show that this can give rise to differential protein expression patterns, using a dual-color in vivo reporter system that simultaneously monitors the cis-regulatory influence of alternative 3′UTR isoforms. In addition, we identify a new developmental program involving coordinated post-transcriptional regulation of nucleolar biogenesis by a conserved interaction between NCL-1/Brat and UUGUU motifs within ribosome-related transcripts.

## Results

### A Quantitative 3′-end Capture Sequencing Method for Low Input RNA Samples

**Fig. 1:**
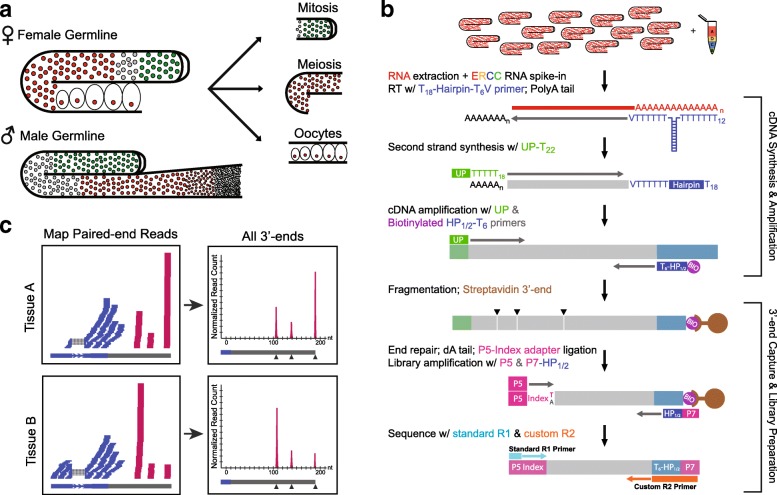
LITE-Seq analysis. **a** Germline tissue used in this study. Whole gonads were isolated from adult hermaphrodite (XX) and male (XO) worms. Female germline samples were generated by manual removal of the spermatheca. Developmentally staged samples were obtained by microdissection of the distal mitotic proliferation zone, the meiotic pachytene region, and cellularized oocytes. **b** LITE-Seq protocol. Extracts of total RNA spiked with ERCC RNA standards were reverse transcribed using an anchored hairpin polydT primer. cDNAs were PCR amplified using a biotinylated half-hairpin primer, fragmented, and subjected to 3′-end capture with streptavidin beads. Samples were barcoded, pooled, and subjected to paired-end sequencing using the standard Illumina Read 1 primer and a custom Read 2 primer that initiates sequencing immediately upstream of the polyA tail. **c** Mapping and quantification of transcript 3′ ends. Paired-end reads were mapped to the *C. elegans* reference genome (WS250) and assigned to genes with overlapping exons or to the closest gene within 1500 nt upstream. Representative 3′ CPA sites (arrowheads) and isoform abundance were determined by clustering 3′ end reads within ±12 nucleotides of the position with the most supported reads

To monitor gene expression and 3′UTR dynamics using small amounts of RNA isolated from manually dissected germline tissue, we developed a new Low-Input 3′-Terminal sequencing method (LITE-Seq) that is both highly sensitive and quantitative (Fig. 1). We isolated mRNA from mixed-stage whole animals, intact gonads dissected from adult males and hermaphrodites with the spermatheca removed (“female” germline), and three distinct regions of the adult hermaphrodite germline: the distal mitotic proliferation zone (partially including the early transition zone), the meiotic region, and developing oocytes (Fig. 1a). These samples allowed us to characterize variation in both mRNA expression and cleavage and polyadenylation (CPA) sites between sexes and as germ cells progress through the germline.

LITE-Seq targets mRNA transcripts using a poly-dT hairpin primer that precisely anchors at the beginning of the poly-A tail (Fig. 1b). First-strand cDNA synthesis is followed by linear cDNA amplification and pull-down using a biotinylated primer that specifically enriches for mRNA fragments containing 3′ends. Reverse transcription is performed using a primer that anchors to the beginning of a polyA tail, so the first base of Read 2 corresponds to the CPA site and thus defines the 3′-end of the transcript. Subsequent paired-end sequencing captures the precise position of CPA sites and produces a quantitative readout of transcript abundance.

To characterize 3′ ends, sequence reads were mapped to a reference genome (Additional file 1: Figure S1) and clustered based on proximity of their 3′ termini to positions supported by the highest abundance of reads, which were designated as representative CPA sites (Fig. 1c). Since the precision of cleavage tends to be tightly distributed downstream of a polyadenylation signal (PAS) sequence in *C. elegans* [43, 44] (Additional file 1: Figure S2A), we clustered CPA sites within ±12 bases of the most highly abundant 3′ read ends (Additional file 1: Figure S3), from which we identified 56,467 representative CPA sites across all samples. Clusters mapping to genomic regions containing downstream polyA stretches and with no identifiable upstream PAS site (8,375 3′ ends) were removed to filter out internal mispriming events, resulting in the final set of 48,092 distinct CPA sites we report in this study.

For comparative analysis between samples, it is crucial to understand the relationship of reads obtained from LITE-Seq with molecular copy number. We used ERCC spike-in standards to confirm a linear relationship between input RNA and read counts (Additional file 1: Figure S4). LITE-Seq provides quantitative readouts with high biological reproducibility for both gene expression (Additional file 1: Figure S5A) and CPA sites (Additional file 1: Figure S5B), and has the added advantage that it captures the precise variation in 3′UTR isoforms across samples.

LITE-Seq shows similar sensitivity to other 3′-end profiling methods. LITE-Seq of germline samples recovered a majority of CPA sites identified in previous surveys [43, 44] (Additional file 1: Figure S6, left) and revealed 17,129 previously unannotated 3′UTR isoforms enriched in specific cell types. The detection rate for CPA sites previously identified in staged whole worm samples correlated with reported transcript abundance (Additional file 1: Figure S6, right), as anticipated from the underrepresentation of somatic tissues in our study and the lower reproducibility of sites with fewer supporting reads. Thus, the LITE-Seq method can detect and accurately amplify low amounts of input RNA to provide quantitative readouts of both transcript abundance and precise locations of cleavage and polyadenylation.

### Gene expression in the germline varies in a sex- and developmentally-specific manner

In total, we observed 14,303 germline-expressed genes: 12,555 in male and 11,394 in female (9,573 in isolated gonads, 8,228 in the mitotic region, 9,008 in the early meiotic region, and 9,067 in developing oocytes). Based on normalized read counts, 8,519 genes were expressed in both sexes, of which 4,929 were present at similar levels (Additional file 2: Table S1). About twice as many differentially expressed genes were present in male (3,338) as in female (1,756) germline, suggesting that spermatogenesis is a highly specialized developmental program.

LITE-Seq compared favorably to several published transcriptome datasets, demonstrating its sensitivity relative to previous studies. We detected 85-95% of germline-enriched genes identified using microarrays [55] (1022/1220 intrinsic germline, 984/1013 oocyte-enriched, and 663/689 sperm-enriched genes) and 85–95% of germline-expressed genes found using RNA-seq [57] (2,415/2,748 of spermatogenic gonad and 1,647/1,732 of oogenic gonad genes). Comparing our data to two recent transcriptome analyses of mature gametes, we recovered 85% (3,175/3,731) of reported sperm transcripts [58], and the majority of reported genes identified in isolated sperm (77%) [59] and oocytes (99%) [27] (Additional file 1: Figure S7). These comparisons indicate that our 3′-end capture method detects gene expression with high sensitivity.

Gene Ontology term enrichment analysis of germline-expressed genes (Additional file 3: Table S2) reflected both shared and distinct biological functions during gametogenesis in the two sexes. A majority of expressed genes were enriched for sexually non-dimorphic functions associated with DNA synthesis, transcription, and translation, evidencing the shared requirements for basic cell biological processes during mitosis and meiosis. Genes more highly expressed in the male germline were enriched for functions associated with phosphatase and kinase activities, as previously reported [58], and the regulation of cell shape, highlighting the importance of post-translational signaling and morphological changes in later stages of spermatogenesis [60]. In contrast, genes with higher expression in the female germline were enriched for functions characteristic of maternal developmental processes: reproduction, embryo development, gene regulation, and metabolism.

Pairwise comparisons of the sectional germline transcriptomes (Additional file 3: Table S2) generally reflected the high biosynthetic activities of the mitotic and meiotic regions, which were enriched for mRNAs with functions related to gene expression and translation relative to oocytes. The meiotic region showed a high level of transcripts related to genome packaging and oogenesis relative to mitosis, and to RNA binding relative to oocytes. The differential expression of transcripts encoding RBPs indicates that the majority are transcribed during pachytene and suggest that at least some of these may be eliminated or deadenylated in oocytes. Taken together, these functional changes reflect the progression of germ cell nuclei through meiosis and the transition to oogenesis and early embryogenesis, which rely heavily on post-transcriptional control by maternal factors.

**Fig. 2:**
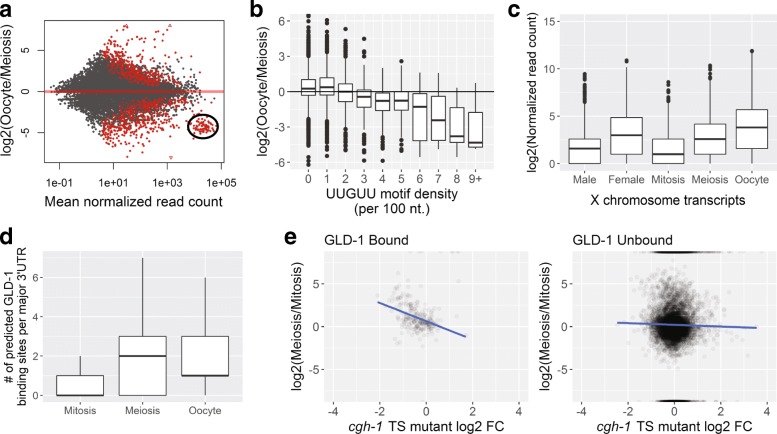
Gene expression dynamics in the germline. **a** Genes expressed at significantly lower levels in oocytes relative to meiosis (circled) or mitosis (Additional file 1: Figure S10) largely comprise ribosomal protein coding genes; rRNA and tRNA biosynthetic genes are also down-regulated in oocytes (Additional file 1: Figure S10). **b** Transcript abundance in oocytes relative to the meiotic region is inversely proportional to the density of UUGUU motifs in 3′UTRs. Oocyte vs. mitosis comparisons are similar (Additional file 1: Figure S11). **c** Expression from the X chromosome is dramatically lower in male germline and in the mitotic region of adult hermaphrodites. X-linked transcripts accumulate in oocytes, but to lower levels than autosomal transcripts, which show relatively even expression throughout the germline (Additional file 1: Figure S8). **d** Major 3′UTR isoforms of genes with significantly higher abundance in meiosis and oocytes relative to mitosis contain a greater number of predicted GLD-1 binding motifs. **e** Differential isoform abundance between oocytes and mitosis (this study) correlates with expression fold-changes of GLD-1 targets in a *cgh-1* temperature-sensitive mutant vs. wild type (data from Scheckel et al. [23]). Our data revealed that fold-changes for GLD-1 targets (left), but not other transcripts (right), correlate with the level of stabilization by CGH-1 observed by Scheckel et al. when comparing expression levels in meiosis (shown here) or oocytes (Additional file 1: Figure S9) relative to mitosis

Genes with higher expression levels in oocytes also showed some notable characteristics. Oocyte-enriched transcripts are depleted for a wide range of biosynthetic functions and are enriched for genes involved in cellular and developmental processes, including signaling, morphogenesis, and nervous system development. These functional trends are consistent with maternal loading of embryos with numerous transcripts required for embryonic patterning, gastrulation, and cellular differentiation [17]. LITE-Seq data also reflect a strong temporal asymmetry in X-linked gene expression (Fig. 2c): transcripts expressed from the X were strongly depleted in mitosis, began to rise in the meiotic region, and increased dramatically in oocytes, but remained lower overall than autosomal expression (Additional file 1: Figure S8). This pattern confirms and extends previous work showing that X-linked genes are transcriptionally silenced in the germline prior to late pachytene, resulting in a short burst of expression before transcriptional activity is shut down globally in developing oocytes [56, 61].

When we looked for highly represented sequence motifs in different samples (Additional file 4: Table S3), the most significant motifs, (A)AURAA, contained the core of the two most abundant PAS sites, which are bound by the cleavage and polyadenylation stimulating factor CPSF (accounting for 48% of all PAS sites). We also observed sequences resembling consensus Puf binding motifs (UGUR core). For example, the mitotic region was depleted of transcripts with binding sites for FBF proteins that were enriched in meiosis (UGUAYW) and oocytes (UGUAHWU) [25], which prevent premature meiotic entry by down-regulating transcripts in the distal germline [24]. Transcripts that accumulate in oocytes were enriched for a polyC motif that directs the degradation of many maternal mRNAs in the embryo shortly after fertilization [27] and for UA[A/U]-rich motifs (UAUDUAU and UAAUUUAU) that could be bound by the oocyte maturation regulators OMA-1 and OMA-2 [62]. Several motifs resemble sites recognized by various other mRNA processing factors involved in transcript biogenesis.

Supporting a major role for spatiotemporal regulation by the translational repressor GLD-1 in the germline, genes with increased expression in the meiotic and oocyte regions contained more GLD-1 binding sites within their major 3′UTRs relative to mitotic cells (Fig. 2d). This timing coincides with the onset of GLD-1 protein expression, which is specific to the pachytene region [19]. In addition to delaying translation of numerous mRNAs until oocyte differentiation or early embryogenesis, GLD-1 is implicated in stabilizing many of these transcripts in conjunction with CGH-1 helicase [23], and our data support this trend in meiosis and oocytes (Fig. 2e and Additional file 1: Figure S9).

In analyzing the dynamics of gene expression, we noticed that a specific subset of highly abundant transcripts in mitosis and meiosis appears to be abruptly cleared in developing oocytes (Fig. 2a, Additional file 1: Figure S10A, and Additional file 5: Table S4). These transcripts encode ribosomal proteins (Additional file 1: Figure S10B), and almost all contain one or more characteristic UUGUU motifs in their 3′UTR. Moreover, the density of UUGUU repeats inversely correlates with their relative depletion in oocytes (Fig. 2b and Additional file 1: Figure S11). In *Drosophila* the UUGUU motif is recognized by Brat, an RNA-binding protein that directs clearance of transcripts containing this motif during the maternal to zygotic transition (MZT) [63]. The *C. elegans* Brat homolog, NCL-1, also binds RNA containing the Brat motif [64]; moreover, *ncl-1* mutant animals are rescued by Brat [65], indicating functional conservation between these proteins in two distantly related animal lineages.

Both NCL-1 and Brat are negative regulators of cell growth, nucleolar size, and ribosomal RNA (rRNA) levels [65–67]. Animals with mutations in *ncl-1* are larger than normal, contain more total protein, and exhibit increased transcription of rRNA genes by RNA Polymerases I and III [67]. Within the adult germline, NCL-1 accumulates to high levels in developing oocytes in parallel with the gradual disappearance of nucleoli [67]. Whereas NCL-1 has no known role in the post-transcriptional regulation of ribosomal subunit genes, NCL-1 and Puf family proteins have recently been implicated as cooperating partners in the translational repression of fibrillarin (*fib-1*), which encodes a ribosomal RNA processing factor [68]. In that study the PUF binding site in the *fib-1* 3′UTR was shown to directly mediate regulation, whereas regulation by NCL-1 was inferred genetically based on increased FIB-1 expression in an *ncl-1* mutant background.

We observe that the *fib-1* transcript contains eight UUGUU motifs (six of which are located in the 3′UTR), and its mRNA abundance decreases around nine-fold in oocytes from its level in the mitotic region. Transcripts encoding several other ribosomal and tRNA biogenesis factors also show decreased expression in oocytes (Additional file 1: Figure S10B) and enrichment for the UUGUU motif in their 3′UTRs – including *pro-1, pro-2, W07E6.2, T04A8.6, K01C8.9,* and *rbd-1* [69]; *dao-5* [70]; and *nol-6* [71]. The concomitant depletion of multiple UUGUU-bearing transcripts and accumulation of NCL-1 protein in oocytes raises the possibility that NCL-1 promotes dissolution of the nucleolus in the -1 oocyte, at least in part, by coordinated translational repression, deadenylation, and/or degradation of transcripts involved in nucleolar biogenesis.

### Identification of 3′UTR isoforms in the germline

LITE-Seq provides data on the precise location of 3′-end processing in mRNA transcripts. To define putative 3′UTR isoforms of protein-coding genes, paired-end reads comprising clustered CPA sites were assigned to genes based on unambiguous overlap with exons (84% of total CPA sites (40,630/48,092)) or, for those with no reads overlapping exons, based on the nearest exon ≤1500 nt upstream (4,634 or 10% of CPA sites). Using this method, we identified a total of 43,725 3′UTRs (91% of CPA sites) for 14,946 protein-coding genes. In addition, we identified 1,485 transcript 3′ ends for 845 annotated non-coding genes and 2,828 that were not near any annotated features and may represent novel unannotated transcripts, which we did not analyze further. After filtering low abundance 3′UTR isoforms (representing <5% of clustered reads per gene) within each sample type, we report a total of 25,450 3′UTR isoforms in the germline.

Around 30% of protein-coding genes in individual samples and 42% cumulatively showed alternative 3′UTR isoforms, revealing tissue-specific selection of 3′UTR isoforms for genes with multiple CPA sites (Additional file 1: Figure S12). The vast majority of 3′-end variation (97%) represented tandem alternative polyadenylation (APA) downstream of the 3′-most CDS stop codon (Additional file 5: Table S4). Annotated functions for genes with multiple isoforms were enriched for protein and nucleic acid binding and for processes involving sexual differentiation and gonadal, embryonic, and larval development. Thus, changes in the germline 3′UTR isoform landscape likely play a role in post-transcriptional regulation of developmental processes.

**Fig. 3:**
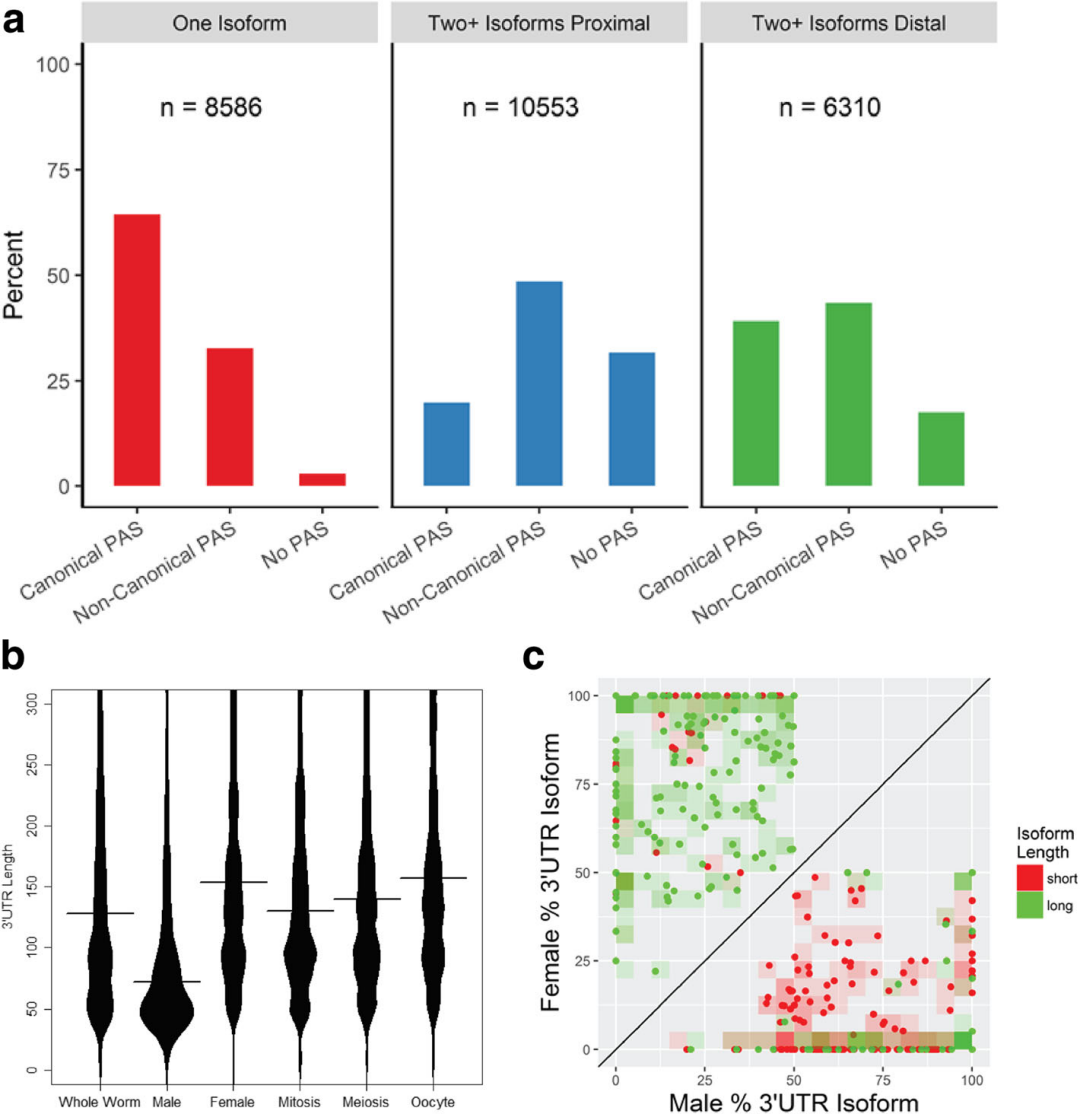
Tissue-specific variation in 3′UTR lengths. **a** PAS site usage for genes with a single or multiple 3′UTRs. The canonical PAS motif (AAUAAA) is the most prevalent and is strongly preferred for genes with a single 3′UTR isoform. **b** Distribution of 3′UTR lengths in different samples. 3′UTRs are dramatically shorter in male vs. female germline. Within the female germline, 3′UTR isoforms are shorter in proliferating mitotic nuclei and longest in oocytes. Highly abundant transcripts for ribosomal and major sperm proteins were excluded from this analysis. **c** Alternative polyadenylation in male and female germline. Genes expressed in both male and female germline show significantly different isoform usage: typically, more proximal isoforms are selected in male and more distal isoforms are selected in female. Each point represents the major isoform for a particular gene, short (red) or long (green), expressed in male (below the diagonal) or female (above the diagonal), along with its relative usage in male vs. female (as percentage of total isoform abundance in each); 5% quadrants are shaded by density of isoform type. Red points in the bottom right quadrant signify that the shortest isoforms for those genes are dominant in the male germline and used less than half the time in the female germline, whereas green points in the top left quadrant indicate the converse (>50% usage of the longest isoform in female and <50% usage in male germline)

Most mRNAs contain polyadenylation signal (PAS) motifs in their 3′UTRs that, together with sequences downstream of the polyA addition site, serve to position the CPA machinery [30]. 81.5% of 3′UTRs contained one of the 15 most common PAS motifs, which were typically located 19 nucleotides upstream of the CPA site [43, 44] (Additional file 1: Figure S13). The majority (61%) of cleavage events for a particular 3′UTR isoform occurred at the same nucleotide position and over 90% of reads were within ±5 nucleotides, though different PAS sequences showed some variation in cleavage precision (Additional file 1: Figure S2B). Genes with a single 3′UTR isoform (8,586) largely use the canonical PAS sequence AAUAAA (64%) (Fig. 3a). For genes with multiple 3′UTRs (6,310), the canonical PAS motif was twice as likely to associate with distal (39%) vs. proximal (20%) CPA sites. Non-canonical PAS motifs constituted a plurality of PAS sites in genes with multiple 3′UTRs, but showed only a marginal preference for proximal (49%) vs. distal (43%) isoforms. In contrast, 3′UTRs with none of the 15 most common PAS motifs occurred at very low frequency among single-UTR genes, and were more often associated with proximal (32%) vs. distal (18%) CPA sites in genes with multiple isoforms. Since we performed stringent filtering of potential CPA sites, we think these results are largely representative of the true distribution of PAS sites. Previous studies also identified a bias toward the canonical PAS motif in distal isoforms and “weaker” CPA signals in proximal isoforms [31, 72].

### The 3′UTR isoform regulatory landscape varies in a cell-type specific manner

3′UTR isoforms were highly dynamic in the germline transcriptome. The distribution of 3′UTR lengths showed large shifts between samples (Fig. 3b), revealing global trends that could significantly impact the tissue-specific cis-regulatory landscape. Overall, 3′UTRs were notably shorter in the male germline, with a median length (72 nt) less than half that in the female germline. These trends remained consistent even excluding the 10% most highly expressed isoforms in each sample and thus are not driven by a small number of short isoforms (Additional file 1: Figure S14). The median 3′UTR length increased as cells progressed through the female germline, from ~130 nt in mitosis to ~157 nt in oocytes. While not extreme, this shift is in line with previously observed trends for proliferating vs. differentiated cells [73].

To gain more insight into potential changes in post-transcriptional regulation, we compared 3′UTR isoforms for individual genes in different cellular contexts. Examining significant changes (FDR < 5%) in the most prevalent 3′UTR isoform, we found that isoform selection for hundreds of genes varied between samples (Additional file 6: Table S5). Of the 7,996 protein coding genes with 3′UTRs common to both male and female germlines, 310 showed a significant difference in isoform usage, with a bias toward proximal isoform selection in males (221/310) (Fig. 3c). While most genes showed the same dominant 3′UTR isoform throughout the female germline, 461 of 9,435 genes expressed in at least two regions showed a shift as gametogenesis progressed (Additional file 1: Figure S15). Usage of proximal or distal isoforms was mixed: roughly half (106/188) of 3′UTRs that switched in length between meiosis and mitosis were longer in the meiotic region; just under two thirds (108/172) were longer in oocytes compared to meiotic nuclei; and about half (157/285) were longer in oocytes vs. mitotic nuclei. Genes whose major isoform changed across germline regions showed no strong bias in functional annotations compared to the full germline transcriptome; however, chromatin organization was overrepresented among genes with both longer (19/157) and shorter (16/128) 3′UTRs in oocytes relative to mitosis, indicating that such transcripts may be important targets for APA and post-transcriptional regulation.

### 3′UTR isoform selection influences protein expression patterns in vivo

**Fig. 4:**
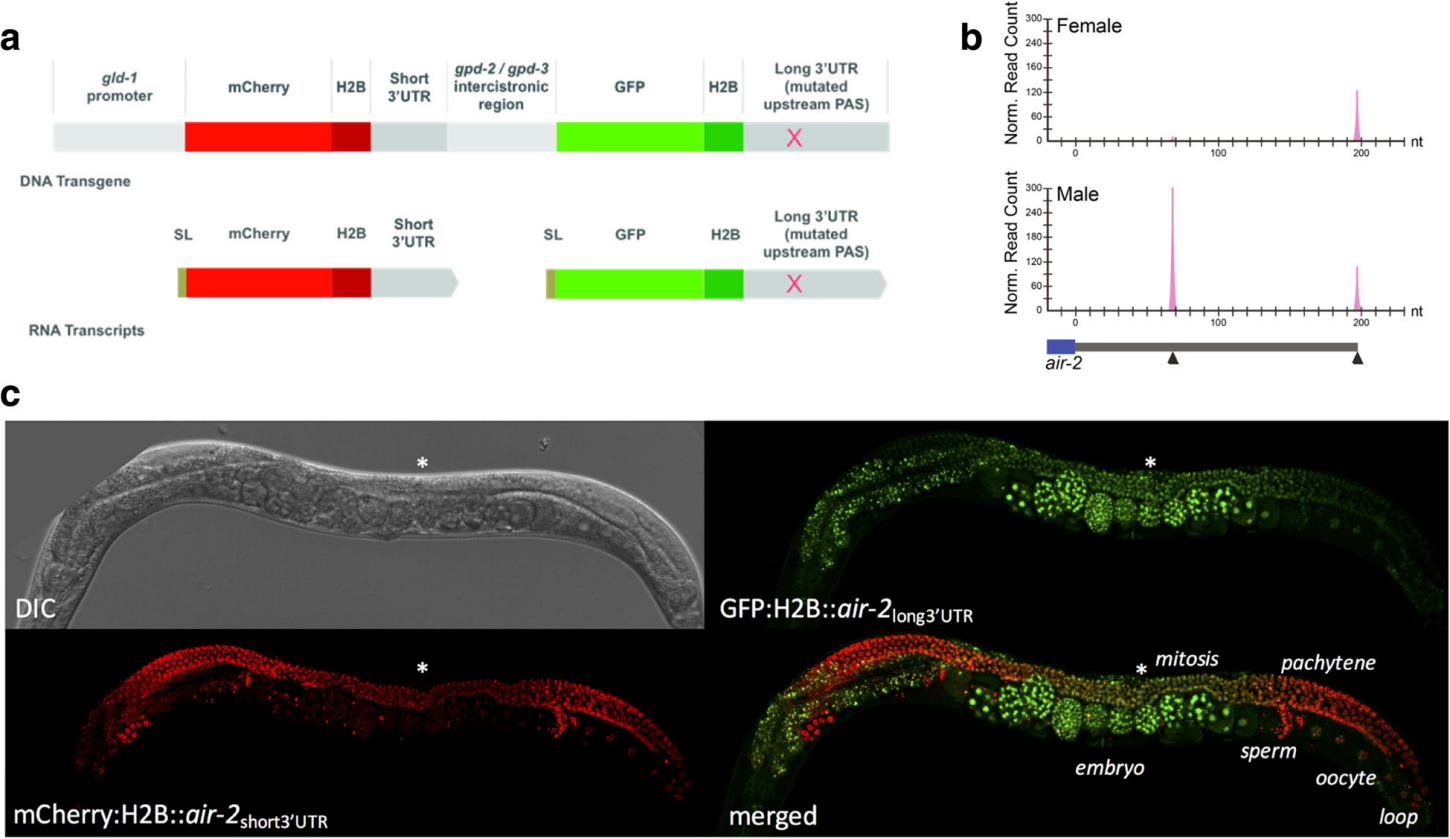
In vivo expression of alternative 3′UTR isoforms in male and female germline. **a** Two-color in vivo reporter system to monitor the influence of different 3′UTR isoforms on protein expression. Fluorescent mCherry and GFP reporters are driven by the same promoter using an operon construct; co-transcriptional processing produces two trans-spliced (SL, splice leader) mRNAs carrying different 3′UTR isoforms. In the long isoform, the upstream PAS site is mutated to ensure usage of the downstream CPA site. **b** LITE-seq evidence for differential alternative 3′UTR isoform usage in vivo. Normalized LITE-Seq read counts for *air-2* CPA sites in different samples. Representative CPA sites defining alternative 3′UTR isoforms are indicated on the gene model below (black triangles). A different 3′UTR isoform is preferred in female (long) and male (short) germline. The short isoform in the male is expressed at around twice the level of the long isoform in the female. **c** Alternative 3′UTR isoforms direct different patterns of protein expression in the female germline. Expression of the mCherry reporter, regulated by the short *air-2* isoform, is similar to controls; GFP expression, regulated by the long isoform, is repressed in pachytene and oocytes relative to controls and reappears robustly in embryos. The distal tip of the germline, where cells begin mitotic proliferation, is marked with an asterisk

To test the idea that alternative isoforms may be differentially sensitive to context-dependent post-transcriptional regulation, we devised an in vivo assay that simultaneously monitors the translational potential of two different 3′UTRs in the germline. We created a reporter system in which mCherry and GFP genes flanked by distinct downstream sequences are transcribed from a single polycistronic operon locus and spliced into individual mRNAs (Fig. 4a, Additional file 1: Figure S16A). Because both reporters are driven by the same promoter, any differences in expression patterns should be dictated exclusively by their 3′UTRs.

Controls with identical 3′UTRs displayed ubiquitous expression in the hermaphrodite germline (Additional file 1: Figure S16C,D), verifying that the *gld-1* promoter is permissive for expression throughout. However mCherry was stronger in mature sperm and also became enriched over GFP in L4 and male germlines as spermatocytes matured. The intensity of both fluorophores in embryos was variable, with the *gpd-2* control 3′UTR appearing to be more permissive than the *rpl-22* control in embryos. Observed biases in expression patterns of control reporters were factored into the interpretation of experimental assays.

As proof of principle, we examined the effect of alternative 3′UTR isoforms on spatiotemporal protein expression using reporters for three genes that showed APA in the germline by LITE-Seq. For all three genes (*air-2*, *aly-3*, and *cap-2*), the major isoform was short in male and long in female germline (Fig. 4b; Additional file 1: Figure S17A), and mCherry (linked to the short 3′UTRs) showed expression patterns similar to controls (Fig. 4c, Additional file 1: Figure S17B,C; compare with Additional file 1: Figure S16C,D). The long isoforms of *air-2* and *aly-3* directed distinct developmental patterns of GFP expression in the hermaphrodite germline that reappeared very robustly in early embryos. Embryonic expression was maternal, as crosses of wild-type hermaphrodites with transgenic males carrying these reporters showed no GFP signal (data not shown). The GFP signal for the Aurora B kinase homolog *air-2* was brightest in the mitotic region, dropped dramatically in pachytene and oocytes, and reappeared in embryos at the one- or two-cell stage (Fig. 4c). This suggests that AIR-2 protein – which functions in chromosome segregation by regulating the release of chromatid cohesion and has an independent role in cytokinesis [74, 75] – is produced during mitosis in preparation for meiosis and is reactivated during mitotic divisions in the embryo. The *aly-3* gene encodes an RRM protein whose homologs recruit mRNA nuclear export factors [76] and can also influence transcription and RNA Pol II occupancy [77]. LITE-Seq detected an *aly-3* transcript with a 205 nt 3′UTR that is a known GLD-1 target [21] and a short (36 nt), previously unannotated 3′UTR isoform lacking the GLD-1 binding motif (Additional file 1: Figure S17A). GFP was brightest in the hermaphrodite mitotic region and attenuated in pachytene relative to mCherry. This pattern is consistent with negative regulation by GLD-1, which accumulates to high levels in pachytene [13, 16, 52]. In addition, GFP expression was strongly suppressed in 1- and 2 cell-stage embryos, reappearing robustly in embryos from the 8-cell stage onward. These results show that the long 3′UTR isoform is needed for repression of *aly-3* in the germline and early embryogenesis. Finally, CAP-2 is the beta subunit of F-actin capping protein and is important for cytoskeletal regulation throughout development [78]. Although *cap-2* isoforms shifted in the female germline, in vivo expression patterns for both reporters were fairly similar to controls (Additional file 1: Figure S17C; compare with Additional file 1: Figure S16C,D).

Collectively, these reporter assays show that forced expression of alternative isoforms can drive different expression patterns in the germline and early embryo, but that some APA events could be benign variants due to cell type-specific differences in 3′-end processing factors. Distinct spatiotemporal patterns indicate that longer 3′UTR isoforms contain negative regulatory sequences that are absent from their short counterparts. While consistent with known examples of translational regulation of maternal products, further work will be required to assess the broader functional consequences of APA in the germline.

### NCL-1/Brat negatively regulates ribosome-related mRNAs through UUGUU motifs

The combined expression dynamics of NCL-1 protein and UUGUU-containing transcripts led us to hypothesize that binding of NCL-1 to this motif contributes to mRNA degradation and/or deadenylation during oogenesis in *C. elegans*. In addition to the large decrease in ribosomal subunit mRNAs (~32-64 fold), we noticed that transcripts for other nucleolar factors, while generally expressed at much lower levels, also decreased by ~2-9-fold in oocytes (Additional file 1: Figure S10B). Coordinated post-transcriptional targeting of ribosomal protein subunits, rRNA processing factors, and tRNA synthetases by NCL-1 could therefore contribute to the rapid disappearance of the nucleolus that is seen in the -1 oocyte.

**Fig. 5:**
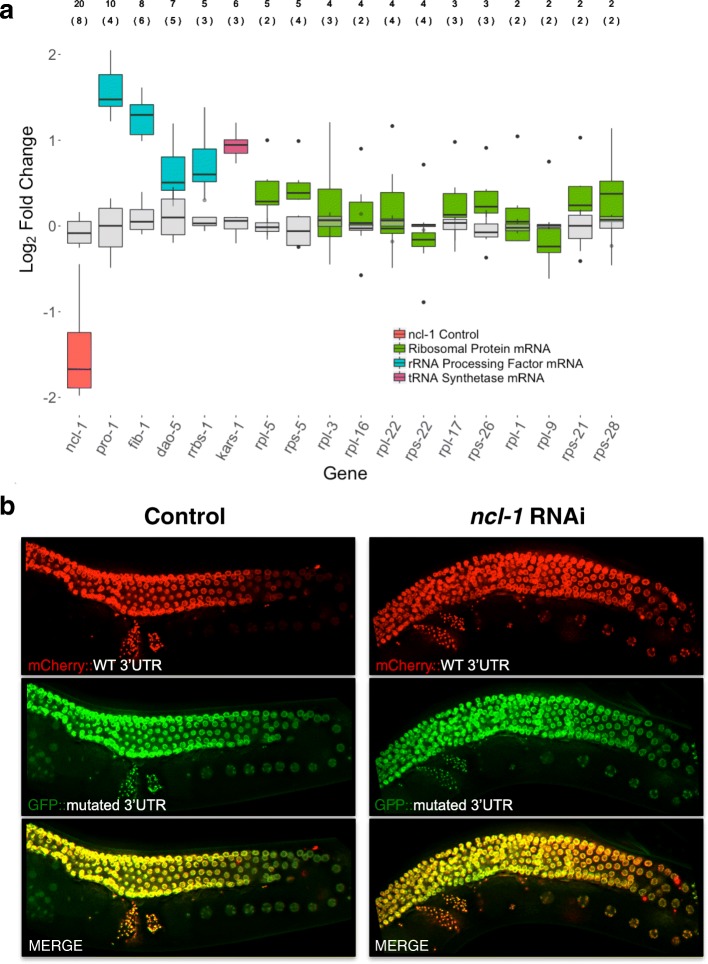
Ribosome-related transcripts containing UUGUU motifs are negatively regulated by *ncl-1* in the germline and embryo. **a** RNAi of *ncl-1* leads to increased expression of ribosome-related transcripts. Log_2_ fold-change in mRNA expression between WT and and *ncl-1*(RNAi)-treated early embryos for ribosome-related genes, as measured by qRT-PCR (n = 8). The median and interquartile range (IQR) for transcripts encoding ribosomal subunits (green), rRNA processing factors (teal), and a tRNA synthetase (purple) upon *ncl-1*(RNAi) (salmon) are shown in comparison with the variation among controls (gray). Whiskers denote 1.5 times the IQR. On average, a 65% knockdown of *ncl-1* mRNA was observed in *ncl-1*(RNAi)-treated worms. Above the graph, the number of UUGUU motifs present in the UTR (top) and in the entire transcript (in parentheses) are indicated. **b** The response of a *pro-1* 3′UTR reporter to *ncl-1* depletion in oocytes is dependent on UUGUU motifs. Representative images of germlines from worms expressing a WT *pro-1* 3′UTR reporter (mCherry) and a reporter fused to *pro-1* 3′UTR containing mutated UUGUU motifs (GFP), fed with empty RNAi vector (control) or *ncl-1* RNAi. Upon *ncl-1* knockdown, mCherry but not GFP expression is increased in the diplotene region and oocytes

To test whether NCL-1 activity influences these mRNAs, we performed quantitative RT-PCR (qRT-PCR) using embryos derived from WT and *ncl-1(RNAi)-*treated animals, reasoning that maternal stores in oocytes should be reflected in early embryos. We found that reducing *ncl-1* levels by around 65% leads to higher levels not only of *fib-1*, but also of several other transcripts related to nucleolar activity, and the effect was proportional to the number of UUGUU motifs contained in these transcripts (Fig. 5a). We also used our two-color in vivo reporter system to test whether 3′UTRs with either intact (UUGUU) or mutated (UUCUU) NCL-1/Brat binding motifs are responsive to varying NCL-1 levels in WT and *ncl-1(RNAi)* germlines. This mutation affects the core of the binding motif [64] and hence should disrupt NCL-1 binding. Again, genes with the largest number of UUGUU motifs in their 3′UTRs (*pro-1* and *fib-1*) showed the greatest difference in expression in oocytes and/or early embryos when comparing WT and *ncl-1(RNAi)* animals (Fig. 5b and Additional file 1: Figure S18A-F). No difference in expression between WT and *ncl-1(RNAi)* was observed for the reporter with mutated UUGUU motifs in the 3′UTR, confirming that these are required for *ncl-1* mediated regulation.

In contrast to the germline LITE-Seq data, which revealed large fold-changes in oocytes for endogenous large (*rpl)* and small (*rps*) ribosomal subunit mRNAs, these showed much more modest and highly variable responses to *ncl-1(RNAi)* in both qRT-PCR (Fig. 5a) and 3′UTR reporter assays (Additional file 1: Figure S18D-F). Thus it would appear that partial knock-down of *ncl-1* is still sufficient to down-regulate these transcripts in oocytes. A logical explanation for this discrepancy, based on stoichiometric considerations, is that the extreme abundance of these mRNAs in meiosis could drive their depletion by NCL-1 as it reaches a very high concentration in oocytes – whereas lower levels of 3′UTR reporter expression driven by the *gld-1* promoter, or partial depletion of *ncl-1* by RNAi, might not be sufficient to elicit a robust post-transcriptional response. Another possibility is that *ncl-1* may cooperate with other factors to regulate these transcripts, as has been shown for *fib-1*. The apparent increase in the GFP-linked *rpl-22* reporter with mutated UUGUU motifs relative to the GFP-linked control *rpl-22* 3′UTR (compare Additional file 1: Figure S18E with Figure S16D and S18F) supports the idea that the motif is necessary but not sufficient for repression when driven at low levels. Notably, the *ncl-1* transcript itself contains 20 UUGUU motifs (8 in the 3′UTR), raising the possibility of a negative NCL-1 self-regulatory feedback loop. Further study will be needed to resolve these outstanding questions.

## Discussion

Here we introduced LITE-Seq, a sensitive 3′-end capture method that simultaneously provides a quantitative measure of gene expression and the precise identification of transcript 3′ ends using limited quantities of RNA. We showed that LITE-Seq accurately reflects isoform-specific expression levels across several orders of magnitude and is highly reproducible, enabling meaningful comparative analyses between different tissues and developmental stages. We used LITE-Seq to characterize the dynamics of gene expression and 3′UTR isoform usage within the *C. elegans* germline and identified numerous examples of alternative 3′UTR isoforms. We demonstrated that naturally occurring 3′UTR variants drive different spatiotemporal expression patterns in vivo and are thus used endogenously to confer differential post-transcriptional regulation in the developing germline and early embryo.

### Germline-expressed genes show sex-specific and spatiotemporal variation

Comparisons of samples between sexes and different developmental zones in the female germline revealed evidence for widespread spatiotemporal variation in both expression levels and 3′UTR isoform selection during developmental transitions in female gametogenesis. Functional annotations revealed large-scale changes in gene expression consistent with known genetic requirements for mitotic proliferation, meiosis, differentiation of male and female gametes, and maternal contributions to early embryogenesis.

Our temporally resolved transcriptome profiling extends previous studies of X-linked expression in the germline. The X chromosome is largely silenced in male and distal hermaphrodite gonads and is activated during a short window in late pachytene and diplotene, before Pol II transcription shuts down in diakinesis [55, 56, 61, 79]. Transcriptome-wide comparisons of LITE-Seq data revealed a small subset of X-linked transcripts that escape silencing in the distal germline; in addition, we found that maximal expression reaches only around half that of autosomal genes, as seen in microarray data for 258 oocyte-enriched genes [61]. X-linked expression in the germline is biased against genes essential for early germline development and toward genes required later during oogenesis or embryogenesis, similar to observations from microarray studies [55].

We investigated mRNA targets of GLD-1, an important post-transcriptional regulator of both entry into meiosis and oocyte differentiation that is expressed at high levels only in the pachytene region [13, 52]. Translational repression of specific transcripts by GLD-1 at this stage is important to delay expression of their protein products until they are needed in developing oocytes or the early embryo, and conversely GLD-1 repression must subsequently be lifted to allow their translation. We observed that transcripts with increased expression in pachytene contained more predicted binding sites for GLD-1 [80], and these were largely retained in oocytes. We also observed a correlation between mRNA levels and the presence of both GLD-1 and CGH-1 binding sites, which have been shown to co-stabilize certain transcripts in association with P granules and P body-like storage granules in the germline [23]. Thus, accumulation of a large body of GLD-1 targets is coordinated with the onset of GLD-1 translation during meiosis, and these transcripts are selectively maintained in oocytes and loaded into the early embryo.

### Post-transcriptional regulation of nucleolar biogenesis reveals a new developmental program in *C. elegans* oocytes

Our germline transcriptome data showed that polyadenylated ribosomal subunit mRNAs abruptly disappear in developing oocytes, and that the vast majority of these contain multiple copies of the short sequence UUGUU. We found that many transcripts encoding rRNA and tRNA biogenesis factors are similarly down-regulated at this stage and also contain UUGUU repeats. This motif is known to mediate maternal transcript degradation by the tumor suppressor Brat during the MZT in *Drosophila* embryos [63]. Brat and its *C. elegans* homolog NCL-1, which also binds UUGUU in vitro [64], are both negative regulators of nucleolar size [65–67], and Brat rescues *ncl-1* mutant phenotypes in *C. elegans* [65].

In the *C. elegans* germline, nucleoli disappear in the -1 oocyte where NCL-1 levels are highest [67], raising the possibility that this is coordinated with oocyte maturation signals from the sperm. Intriguingly, mouse oocytes also show a decrease in ribosomal protein transcripts and total rRNA as part of a broad mRNA decay program that takes place during meiotic maturation [81, 82]. Thus, the suspension of ribosomal biogenesis in preparation for the completion of meiosis upon egg activation appears to be conserved between nematodes and mammals and represents a previously unrecognized developmental event in the *C. elegans* oocyte-to-embryo transition. This regulatory program precedes the MZT in *C. elegans* [26] and is independent of the poly-C cis-regulatory motif that contributes to maternal mRNA turnover in the fertilized zygote [27].

Our data provide a new angle on how NCL-1 likely regulates nucleolar activity. Prior to this study knowledge of *ncl-1* function was restricted to genetic evidence that it works together with Puf family proteins to repress translation of a single transcript, encoding the rRNA processing factor fibrillarin (FIB-1) [68]. However, *fib-1* regulation cannot fully account for the nucleolar functions of *ncl-1*, as evidenced by the fact that *fib-1* knockdown does not rescue the increase in nucleolar size observed in *ncl-1* mutants [68]. Our data provide a molecular basis for this and indicate that NCL-1 acts in a much broader fashion to negatively regulate dozens of genes related to nucleolar biogenesis through direct binding of UUGUU motifs present in these transcripts.

In the early embryo, *ncl-1* phenotypes signal that ribosome biogenesis is prematurely activated: DAO-5 precociously localizes to nuclear spots at the 2-cell stage [83], and rDNA transcription and enlarged nucleoli are seen at the 4-cell stage [67]. The up-regulation of ribosome-related transcripts that we observed in oocytes and embryos upon *ncl-1* depletion implies that nucleolar phenotypes arise from defects in deadenylation and/or degradation of maternal transcripts. Since *ncl-1* mutants appear to have no effect on transcription by RNA Pol II [67], a post-transcriptional mechanism that broadly targets ribosomal proteins, rRNAs and tRNAs would provide an efficient mechanism to simultaneously shut down multiple facets of nucleolar biogenesis, in turn leading to reduced rRNA transcription and nucleolar size [67]. Deciphering its primary mechanism of action awaits future studies of its effects on endogenous mRNA targets to help clarify whether it binds cooperatively to multiple motifs or with other factors, and how this may influence mRNA translation, deadenylation, and/or stability.

It is interesting that NCL-1 itself is developmentally regulated, showing lowest levels in cells with higher metabolic activity, such as the intestine [67]. The fairly low levels of *ncl-1* mRNA throughout the germline (around 80-100 counts) could be maintained by negative self-regulatory feedback, as it contains 20 NCL-1 binding motifs (the most we found in any germline transcripts). The transcript must also be under translational control in the germline, as it accumulates to extremely high levels in oocytes whereas the mRNA does not. Its 3′UTR additionally contains a functional binding site for the heterochronic miRNA *let-7* [68], providing a potential mechanism to coordinate its activity with developmental growth cycles. As nucleoli link overall biosynthetic capacity with the cell cycle, cell growth, nutritional status, and stress response [84], it is tempting to speculate that NCL-1 may be a master regulator of nucleolar function that senses metabolic and developmental cues and responds by titrating global biosynthetic activity to promote cellular homeostasis at all stages of development.

### The 3′UTR isoform regulatory landscape varies in a cell-type specific manner

Analysis of our sequencing data highlights the advantages of the LITE-Seq method for identifying and quantifying expression of distinct 3′UTR isoforms for any given gene. By analyzing global dynamics of the 3′UTR landscape, we found that 40% of germline-expressed genes are alternatively polyadenylated, and we uncovered numerous 3′UTR isoforms not identified in previous studies. We found that most cleavage and polyadenylation sites are very sharp, i.e. individual transcripts show 3′ ends that cluster tightly around a preferred position. Similar to previous studies [43, 44], we observed that *C. elegans* uses a wide variety of signal sequences. Whereas in humans the canonical PAS is strongly preferred in all 3′UTRs [85], *C. elegans* seems to have much looser requirements, particularly in genes that undergo alternative polyadenylation. This suggests either that the CPA machinery is less stringent or that additional sequence features and/or cleavage factors confer specificity to these sites. Comparisons of 3′UTR usage between different samples allowed us to identify numerous genes that show differential APA between sexes and different stages of germ cell development. Given the widespread nature of APA in the germline, it appears to be regulated in a cell-type-specific manner to ensure the inclusion or exclusion of specific cis-regulatory motifs. We cannot exclude the possibility, however, that some of the observed variation in transcript termination is a benign byproduct of cell-type-specific differences in transcriptional activity or the complement of mRNA processing factors, which can all contribute to the global APA profile [86].

The length of 3′UTRs was generally much shorter in males, as also observed in *D. melanogaster* [37]. This suggests that the spermatogenic program may rely more heavily on transcriptional regulation, though some key events depend on translational control [87]. In *C. elegans*, the shift to shorter 3′UTR isoforms may potentially be linked to the RNA cleavage factor CFIm, which upon knockdown in mammals causes preferential usage of proximal sites [88]. We detected significantly lower expression of CFIm-1 and CFIm-2 in the male germline, indicating that this may be an operant factor in the bias toward shorter 3′UTRs. Mammals also show testis-specific patterns in APA and expression of CPA factors [89]. Notably, 3′UTRs undergo progressive shortening during mouse spermatogenesis, and this is associated with changes in chromatin states and transcriptional activity [90, 91]. Shortened 3′UTRs tend to exclude destabilizing elements – such as U-rich elements – and lengthened 3′UTRs add sequences that may help stabilize transcripts [91]. The similarity of trends across distantly related species suggests the intriguing possibility that regulated APA selection favoring shorter 3′UTRs may be a deeply conserved property of male germline development.

Spatiotemporal control of translation and mRNA stability plays an important role in both oogenesis and embryogenesis and is often achieved by the coordinated action of multiple RBPs [5, 8, 16, 29, 92]. Because it allows for change on a shorter timescale than transcriptional control, post-transcriptional regulation is thought to facilitate the rapid developmental transitions that occur in this complex tissue during gametogenesis. While certain key events in the distal germline, including the mitosis to meiosis transition, are controlled by 3′UTR-mediated interactions [16, 18], we observed a gradual increase in average 3′UTR length during germ cell development in the female. Thus it would appear that post-transcriptional control is more broadly employed during later stages of oogenesis, consistent with well-documented genetic requirements for translational regulation of maternal products during oocyte development and early embryogenesis [16, 29]. Transcripts with shorter 3′UTR isoforms may tend to be translated more efficiently, since many RBPs act as translational repressors at these stages [5, 17]. The regulated production of multiple isoforms may thus allow expression levels of individual proteins to be tuned depending on the specific needs of cells at different stages of development.

### 3′UTR isoform selection directs differential protein expression patterns in vivo

3′UTRs have been shown to be the main drivers of spatiotemporal protein expression patterns for numerous genes in the germline [14]. To test the biological effects of endogenous 3′UTR variants that we identified, we examined patterns of reporter gene expression in vivo for three genes whose dominant isoforms differ between sexes or in different regions of the female germline. For two genes, reporters for short and long 3′UTR isoforms displayed different localization patterns when driven by a ubiquitous germline promoter. Unlike their shorter counterparts, long isoforms experienced negative regulation in the female germline and showed reactivation of expression in early embryos. Thus, selective inclusion of specific post-transcriptional regulatory elements in longer 3′UTRs can allow for more nuanced patterns of regulated expression in the germline and early embryo.

More generally, the observed sex-specific and spatiotemporal differences in isoform prevalence and protein expression driven by alternative 3′UTRs point to isoform selection as a functionally important feature of both the spermatogenic and oogenic differentiation programs. Post-transcriptional regulation is specified by a combination of RNA-binding proteins and small RNAs and is achieved through selective translation, stabilization, deadenylation, and/or degradation of mRNAs bearing alternative 3′UTR isoforms. Since LITE-Seq detects only polyadenylated transcripts, future studies will be required to distinguish between these alternative mechanisms in the germline. This work sets the stage to learn more about how cytoplasmic and nuclear processes such as epigenetics, transcription, splicing, APA, and post-transcriptional regulation are integrated to govern the lifecycle of mRNAs that drives patterned gene expression during development.

## Conclusions

We have developed a new experimental method, LITE-Seq, which quantifies the 3′ends of all polyadenylated mRNAs expressed in vivo using small amounts of input sample. LITE-Seq provides a new tool to probe 3′UTR variation in different cellular and developmental contexts. Spatiotemporal profiling of the *C. elegans* germline with LITE-seq revealed dynamic changes in both the expression and diversity of 3′UTRs during gametogenesis at unprecedented resolution. Nearly half of germline-expressed genes are alternatively polyadenylated, suggesting that differential post-transcriptional control is pervasive in the developing germline. Shorter transcripts are expressed in males and in rapidly proliferating mitotic cells, while longer transcripts expressed during meiotic differentiation in the female germline expose a greater repertoire of putative target elements for trans-acting factors. The transcripts expressed later in gametogenesis, which are expected to be under more complex regulatory control, encode proteins involved in gene regulation, development and morphology. We show that alternative 3′UTRs can drive distinct protein expression patterns across the male and female germline, demonstrating isoform-specific regulation of endogenous transcripts. The broader functional significance of these differences awaits further study.

We discovered a post-transcriptional developmental program that down-regulates mRNAs involved in ribosome, rRNA, and tRNA biosynthesis in oocytes, coincident with the dissolution of the nucleolus just prior to fertilization. We demonstrate that the RBP NCL-1/Brat is a regulator of this transition and show that it acts through UUGUU motifs present in these transcripts. We propose that NCL-1 acts as a master regulator of the nucleolus by promoting the coordinated down-regulation of a broad spectrum of transcripts related to ribosomal biogenesis and nucleolar function.

In summary, a careful analysis of gene expression dynamics coupled with the precise 3′UTR landscape provides a sensitive and quantitative spatiotemporal map of gene expression and 3′UTR variation within the *C. elegans* germline. The analysis reveals widespread alternative polyadenylation and the differential regulation of transcript levels, isoforms, and translational potential during germ cell development. In addition, we identify a previously unrecognized developmental program that directs disassembly of the nucleolus prior to completion of the meiotic divisions through post-transcriptional regulation. We conclude that selective regulation of 3′UTR sequence content is an endogenous mechanism used in this complex tissue that allows for cell type-specific control of mRNA fates, and that this contributes to key developmental transitions during *C. elegans* gametogenesis and early embryogenesis. The widespread and dynamic 3′UTR diversity we observe offers a rich source of regulatory potential in germline development. Ongoing systems approaches aimed at combining isoform-specific expression with chromatin states, mRNA synthesis, and protein-RNA interactions should help arrive at a holistic view of how sex- and spatiotemporally-specific trans-acting factors integrate these levels of regulation into dynamic developmental programs.

## Methods

### *C. elegans* worm strains

The wild-type N2 Bristol strain was used for all sequencing experiments. Worms were maintained at room temperature on NGM-agar plates seeded with *E. coli* strain OP50-1. N2 males were generated by heat-shocking L4 hermaphrodite worms at 30 °C for 6 hours to induce nondisjunction of the X chromosome and were maintained by continuous crossing. Transgenic lines were created using the EG6699 strain for MosSci [93] insertions onto chromosome II and maintained at 25 °C on NGM agar plates seeded with *E. coli* strain HB101. Transgenic worms expressing the two-color reporter constructs were generated at Knudra Transgenics (Murray, UT) or injected in our lab as described in [93] using the MosSCI strain EG6699. Constructs were injected into young adults and recovered at room temperature. Plates containing rescued Unc worms were screened seven days later for the absence of negative selection markers. Worms were passaged to new plates, and homozygotes analyzed for expression of mCherry and GFP in the germline. Additional file 7: Table S6 lists all transgenic strains constructed for this study.

### RNA sample collections for LITE-Seq

Worms were synchronized by plating arrested L1 larvae and allowed to grow to young adults. N2 hermaphrodites from non-mated plates or N2 males were dissected in a depression slide containing 0.5 mM levamisole in M9 buffer. Heads were severed below the pharynx with a 25G1½ needle, and intact extruded germ lines were separated from the body. The ‘female’ germline was cut to include everything except the spermatheca, while the male germline was cut to include spermatids, although some were released into the buffer when dissected. Germline pieces were visually assessed and cut under dissecting microscope. Samples were immediately immersed in RNALater buffer (Qiagen, Germantown, MD) to preserve the RNA and then transferred using a mouth pipet to the cap of a 0.2 mL microcentrifuge tube containing additional RNALater. Samples were pooled in the same cap until sufficient quantities were collected and then stored at -80 °C. Once samples had been collected in triplicate, RNA was extracted using the Qiagen RNeasy Micro kit according to the manufacturer’s protocol with an on-column DNase I treatment and eluted in RNase-free water. The quantity and quality of RNA recovered were assessed on an Agilent Bioanalyzer (Santa Clara, California).

### cDNA Amplification, Library Preparation and Sequencing

To generate cDNA, we used 65 ng or 50 ng of total RNA from each sample pool. The ERCC RNA spike-in mix (Invitrogen, Carlsbad, California) was added in quantities recommended by the manufacturer. RNA was added to lysis mixture (0.9X PCR buffer, 3 mM MgCl2, 0.45% NP40, 4.5 mM DTT, 0.18U/ul SUPERase-In, 0.36U/ul RNase Inhibitor, 0.125uM dNTP) and heated to 70 °C for 90 seconds. First strand cDNA synthesis was performed using SuperScript III reverse transcriptase (Invitrogen) along with a modified poly-dT primer in reactions containing 13.2U/ul SuperScript III, 0.5uM poly-dT hairpin primer, 0.4U/ul RNase Inhibitor, and 0.07ug/ul T4 gene 32 protein (Invitrogen). The primer (5′-T_18_TGGAATTCTCGGGTGCCAACCCTTGGCACCCGAGAATTCCAT_6_V-3′) contains a single non-T nucleotide at the 3′ end, which serves to anchor it to the transcript immediately upstream of the polyA tail, and an internal sequence that forms a hairpin loop. Prior to the RT reaction, the primer was heated to 98 °C for 10 minutes and allowed to slowly cool to room temperature in order to ensure proper formation of the hairpin. Following first strand cDNA synthesis, ExoSAP (Affymetrix, Santa Clara, California) was added to remove all remaining free primer. The cDNA was polyadenylated using a terminal transferase and treated with RNaseH to remove the RNA strand (New England Biolabs, Ipswitch, Massachusetts). Purification of the single-stranded cDNA was done using Ampure beads (Agencourt, Lynn, Massachusetts). The second strand of cDNA was synthesized using a UP2-polydT primer (5′-ATATCTCGAGGGCGCGCCGGATCCT_22_-3′) and Takara Taq polymerase (Nojihigashi, Japan). Eighteen rounds of cDNA amplification were performed using a UP2 primer (5′-ATATCTCGAGGGCGCGCCGGATCC-3′) and a biotinylated primer containing only a part of the hairpin sequence (5′-Bio-CAACCCTTGGCACCCGAGAATTCCAT_6_-3′) in order to limit residual polyA tail sequences to 6 nt in the final product. Half of the cDNA was re-amplified by another six cycles of PCR to generate sufficient cDNA for library preparation, repurified with Ampure beads, and examined on the Bioanalyzer to assess quality and quantity of the PCR products.

For library preparation, approximately 1ug of amplified cDNA was fragmented in a S220 Focused Ultrasonicator (Covaris, Woburn, Massachusetts). Fragments between 300–400 bp were selected with a gel-free size-selection protocol using Ampure beads. Streptavidin beads (Invitrogen) were added to capture biotinylated cDNA ends corresponding to the original 3′ ends of the transcript. 10ul of streptavidin beads were washed and resuspended in 20ul of 2x binding buffer (10 mM Tris pH7.5, 1 mM EDTA, 2 M NaCl, 0.02% Tween20). While still attached to beads, the captured cDNA was end-repaired and an adenosine was added to the 3′ end (NEBNext Ultra End Repair/dA-Tailing module). Illumina P5 adapters carrying a six base barcode were T/A-ligated to the free end of the DNA fragments (5′-AATGATACGGCGACCACCGAGATCTACACTCTTTCCCTACACGACGCTCTTCCGATCT-XXXXXX-T-3′). The library was amplified from the beads for twelve cycles with KAPA HiFi HotStart ReadyMix (Kapa Wilmington, Massachusetts) using a P5 primer (5′-AATGATACGGCGACCACCGAGATCT-3′) and a P7-half-hairpin primer (5′- CAAGCAGAAGACGGCATACGAGATCCAACCCTTGGCACCCGAGAATTCCA -3′). During PCR amplification, the beads easily settle to the bottom. To ensure proper amplification of the library, the tubes were quickly vortexed after each denaturing step. Libraries were cleaned with Ampure beads and checked on a Bioanalyzer for quality and quantity before storage in −20 °C. Prior to sequencing libraries were quantified using the KAPA Library Quantification Kit, diluted to a concentration of 2nM, and pooled. A PhiX library was added at approximately 20% to increase the base diversity. A paired-end 100 bp rapid run was performed on the Illumina HiSeq 2500 platform (San Diego, California) using the standard Read 1 primer and a custom sequencing primer for Read 2 (5′-AGCAGAAGACGGCATACGAGATCCAACCCTTGGCACCCGAGAATTCCAT_6_ -3′), which is designed to provide sequences starting from the last base prior to the poly(A) tail.

### Bioinformatic Analysis

Sequencing reads were de-multiplexed into sample libraries according to the barcode encoded in the first six bases of Read 1. Reads were trimmed of the barcode and Illumina adaptor sequences. Short (<50 nt) or low quality reads (≤95% identity) were excluded from further analysis. Reads were mapped to the *C. elegans* reference genome (WS250) using paired-end reads with TopHat 2 in an iterative manner with parameters “--b2-very-sensitive --min-segment-intron 14 --min-coverage-intron 14 --min-intron-length 14 --max-segment-intron 24000 --max-coverage-intron 24000 --max-intron-length 24000 --read-edit-dist 5 --read-realign-edit-dist 0 --segment-mismatches 3 --read-mismatches 5 --max-deletion-length 5 --max-insertion-length 5 --no-discordant”, mate-inner-distance and mate-std-dev were determined using Qualimap 2 [94]. Read 2 of unmapped paired-reads were remapped after trimming any stretches of thymidines at the beginning of the sequence, which occasionally occurred due to mis-anchoring of the hairpin primer within polyA tails during the RT reaction. Any mapped reads potentially generated by internal priming of the hairpin primer to A-rich regions within transcripts (i.e. that matched genomic sequence and thus most likely did not represent polyA tails) were filtered. Finally, remaining unmapped Read 2 sequences were remapped after trimming to 50 nt to remove occasional palindromic sequences that were occasionally observed among the unmapped reads and which presumably arose from technical errors during PCR amplification. Any CPA sites for which Read 2 began with an adenosine, was followed by 3 adenosines in the next 4 bases, and did not contain a known PAS site 8–25 nucleotides upstream of the putative CPA site were removed as potential false positives; those containing a PAS site were retained as a separate set of potential true positives but were not included in our final analysis. Raw and mapped reads were deposited at NCBI Sequence Read Archive, accession number SRP096640.

We assigned read-pairs to an annotated gene if reads overlapped a known exon using HTSeq [95] or, if no exons overlapped the read, to the closest annotated gene within 1,500 nucleotides upstream. To identify differentially expressed genes, libraries were first normalized using the size factor calculation from DESeq2 [96] to allow comparisons between libraries. GO term enrichment analysis for differentially expressed genes was performed using PantherDB [97].

In order to define 3′UTR isoforms detected within each sample, a ‘universe’ of all possible cleavage and polyadenylation (CPA) sites was established by combining Read 2 data from all 14 libraries. Reads starting at a given position (chromosome and strand) found in at least two samples were summed and sorted in descending order of abundance, if a position was not supported by at least one read in two samples, read counts supporting this position were not included. CPA sites were defined by clustering reads within a ±12 nucleotide window of the position with the highest number of supporting reads. If two sites within a given window were supported by the same number of reads, we selected the 3′ most position.

To determine 3′UTR isoform switching between samples, potential CPA sites were identified for each gene in every sample. Major isoforms with the highest usage were identified in each sample. Genes that switched usage from one major 3′UTR isoform to another in a given set of samples were identified using an algorithm for calculating Significance Analysis of Alternative Polyadenylation [98]; to allow for cases with more than two isoforms, relative expression for each given isoform was compared to collective usage of all other isoforms.

### Two-color Reporter System

Reporter constructs were made in several steps using the MultiSite Three-fragment Gateway cloning system (Invitrogen), as illustrated in Additional file 1: Figure S16. Additional file 7: Table S6 lists all primer sequences and plasmids used for cloning and resulting vectors. To generate the final reporter constructs for each pair of 3′UTR isoforms, three Entry clones were generated as described below and combined together with LR+ clonase into Gateway Destination vector pCFJ150 for MosSCI recombination.

Entry Construct 1, containing the *gld-1* promoter, was PCR amplified from *C. elegans* N2 genomic DNA using primers DM126 and DM127. The cassette spans 530 bp of genomic DNA, beginning immediately downstream of the T23G11.4 CDS stop codon (DM126) and ending with the *gld-1* CDS start codon (DM127). The PCR product was recombined into pDONR P4-P1R using BP clonase (Invitrogen).

Entry Construct 2, the operon-reporter cassette, contains the *gpd-2/gpd-3* intergenic region from the *mai-1* operon [99] flanked by GFP:H2B and mCherry:H2B reporters. The operon cassette was made in several steps using a pDONR 221 vector modified to include part of the multiple cloning region (MCR) from pSL1180 (Addgene, Cambridge, MA) that spans 17 restriction sites from SpeI to BglII. A 244 bp fragment containing the entire *gpd-2/gpd-3* intergenic region was PCR amplified from N2 genomic DNA with primers DM144 and DM145. The genomic sequence begins immediately downstream of the *gpd-2* CDS stop codon (DM144), includes the full 133 nt *gpd-2* 3′UTR, and ends immediately upstream of the *gpd-3* CDS start codon (DM145). The resulting PCR product was cloned between the SacII and NotI sites in the MCR. Fluorescent reporter genes were then cloned between SpeI-SacI (upstream gene) and NotI-BglII (downstream gene) sites. We generated constructs containing GFP:H2B and mCherry:H2B reporters in both orientations (i.e. mCherry upstream and GFP downstream, and vice versa). GFP::H2B and mCherry:H2B were amplified from plasmids pGC468 and pGC544, gifted by the Nance laboratory, using primers containing the corresponding restriction sites flanked by gene-specific sequences.

For gene-specific 3′UTR reporter constructs, short and long 3′UTR isoforms were PCR amplified from N2 genomic DNA using primers targeting the regions of interest and flanked by appropriate cloning sites (described below). For reporters with UUGUU motifs, WT and mutated (UUGUU to UUCUU) 3′UTR oligos were chemically synthesized (IDT). Short 3′UTR isoforms and WT UUGUU 3′UTRs were cloned into Entry Construct 2 between SacI and SacII to generate gene-specific variants. Cassettes with long 3′UTR isoforms and 3′UTRs with mutated UUCUU motifs flanked by Gateway sites were used to generate a gene-specific Entry Construct 3 in pDONR P2R-P3. To ensure correct CPA site selection for long 3′UTR isoforms, any upstream PAS sites were eliminated using site-directed mutagenesis. PAS sites were altered by 25 cycles of “around-the-world” PCR with Phusion polymerase (New England Biolabs), using complementary primers containing mismatches within the motif and flanked by perfect matches of 20 nt upstream and downstream. PCR products were cleaned, concentrated, and then digested with DpnI to degrade the original template.

### Quantitative RT-PCR

To synchronize animals for RNAi treatment and RNA extraction, gravid N2 worms were bleached, released embryos were grown overnight in M9, and ~25,000 L1 larvae were plated onto two extra large NGM-IPTG plates seeded with *E. coli* containing either *ncl-1* or empty control (L4440) RNAi vectors. These plates were left at 20 °C until the worms were gravid and some embryos were visible on the plate. Several worms from each experimental plate were checked for *ncl-1* RNAi phenotypes (e.g. presence of a nucleolus in the -1 oocyte). Once the presence of these phenotypes was confirmed (Additional file 1: Figure S18G), the worms were bleached and any remaining carcasses removed via vacuum filtration. Aliquots of bleached embryos were examined under 100x magnification to ensure a good representation of early embryos (1-64 cells). Total RNA was extracted from these embryos using RNeasy Mini Kit (Qiagen), and any remaining DNA was digested with EZ DNAse (ThermoFisher, Waltham, MA). Each collection yielded ~40-50ug of RNA. 20ug of RNA was then reverse transcribed into cDNA using Superscript IV reverse transcriptase (ThermoFisher). qPCR was performed on a LightCycler 480 (Roche, Indianapolis, IN) using LightCycler 480 Sybr Green 1 Master (Roche). Results were analyzed using the ΔΔC_T_ method [100]. For each gene, eight independent biological replicates were assayed using two technical replicates per assay. For comparison, variation among control replicates was calculated by normalizing to the mean of the replicates and calculating the log_2_ fold-change.

### Microscopy

Z-stacks spanning approximately 20um were acquired at 20x or 40x with a Leica DFC365 FX digital camera attached to a Leica DM5500 B microscope (Leica, Wetzlar, Germany) using filters for GFP (Ex:450-490, DC:495, EM:500-550) and mCherry (Ex:540-580, DC:595, EM:607-693) and DIC optics for bright field. Images were processed by deconvolution and maximum projection of the relevant Z stacks using LAS X (Leica).
